# Identifying plastics with photoluminescence spectroscopy and machine learning

**DOI:** 10.1038/s41598-022-23414-3

**Published:** 2022-11-06

**Authors:** Benjamin Lotter, Srumika Konde, Johnny Nguyen, Michael Grau, Martin Koch, Peter Lenz

**Affiliations:** 1 Department of Physics, Philipps-Universität Marburg, Marburg, Germany; 2 Department of Medicine A, Hematology, Oncology, and Pneumology, University Hospital Münster, Münster, Germany

**Keywords:** Environmental impact, Marine chemistry, Pollution remediation

## Abstract

A quantitative understanding of the worldwide plastics distribution is required not only to assess the extent and possible impact of plastic litter on the environment but also to identify possible counter measures. A systematic collection of data characterizing amount and composition of plastics has to be based on two crucial components: (i) An experimental approach that is simple enough to be accessible worldwide and sensible enough to capture the diversity of plastics; (ii) An analysis pipeline that is able to extract the relevant parameters from the vast amount of experimental data. In this study, we demonstrate that such an approach could be realized by a combination of photoluminescence spectroscopy and a machine learning-based theoretical analysis. We show that appropriate combinations of classifiers with dimensional reduction algorithms are able to identify specific material properties from the spectroscopic data. The best combination is based on an unsupervised learning technique making our approach robust to alternations of the input data.

## Introduction

Plastic pollution is considered one of today’s main environmental problems. With worldwide production rates of 460 million tons per year and recycling rates being only at 9%, more and more plastic debris is ending up in the environment. Significant amounts of plastics have already accumulated in aquatic environments (109 million tons in rivers and 30 million tons in the ocean)^1^. This number continues to grow as the annual worldwide production rate of plastics is continuously increasing and even outpacing economic growth. Unfortunately, plastics can persist for decades as most types are resistant to natural degradation processes^2^.

Only under harsh environmental conditions, such as extensive exposure to sunlight, plastics can fragment into micrometer-sized particles commonly known as microplastics^3–8^. Being almost invisible to the eye, the potential harm of microplastics has been ignored for decades. However, this is beginning to change as we now find them in almost every corner of our planet^7,9–13^, in animals^14–16^, and in our food^17,18^. Most recently, microplastics was found in the placenta^19^ and in our blood^20^, which shows that plastic litter has finally found its way into our bodies^21–23^. These alarming findings urge us to increase our efforts to track the fate of plastic litter in our environment and to implement worldwide effective waste management plans to avoid further plastic litter accumulation^24–26^.

Such strategies have to be based on a quantitative understanding of plastic litter distribution and characterization. To obtain the necessary data, plastic litter monitoring has to be implemented in combination with the use of consistent and reliable methods of sample characterization^27^. In particular, valuable insights about average microplastic size and material type and their global distribution can be gained by analyzing plastic samples extracted from different sites. The material type is highly relevant for plastic pollution mitigation, as it could allow us to pin down site-specific sources for the most common plastic litter types. Detecting plastics in our environment, however, is a challenging task, as they are diverse: during production, additives are frequently utilized to change the material properties of plastics. In the environment, these properties can change and deteriorate after long exposure times. Therefore, it is fundamental to conduct frequent and consistent sampling at multiple sample sites.

There are several detection techniques available that can be used to identify plastic litter^28,29^. Generally, non-destructive spectroscopic techniques should be preferred as they allow us the cross-validation with other analytical tools. Such techniques probe the sample with a light source and measure the emitted light with a spectrometer to acquire a spectrum. The interaction of the sample with light depends on the sample’s chemical composition implying that a spectrum can contain information similar to a fingerprint for sample material identification. Studies on plastic pollution commonly use solutions based on Raman spectroscopy or Fourier-transform infrared (FTIR) spectroscopy to analyze plastic samples^29–32^. However, both techniques come with physical limitations^30,33,34^ obstructing the detection of several types of plastic litter.

Recently, Ornik et al. have demonstrated that photoluminescence (PL) spectroscopy can be used for plastic litter identification^35^. The big advantage of this technique is its simplicity. A set-up consists of a light source that emits monochromatic light in the visible range, a spectrometer, and a set of lenses to collect the light emitted from the sample. Since the amount of necessary components is lower compared to Raman and FTIR spectroscopy, the acquisition costs for PL spectroscopy are lower. As a result, it should be ubiquitously accessible compared to the aforementioned techniques. This, in turn, can be used to systematically extend plastic litter monitoring by conducting sampling worldwide and will allow us to establish databases of spectral data sets capturing sample diversity.

The prediction of sample properties like material type from its observed spectrum is based on modeling spectral features such as intensities at certain wavelengths. For PL spectroscopy, we have shown that most common plastic types can be distinguished from non-plastic samples from the marine environment, simply by comparing certain spectral intensity ratios between different samples^35^. However, such a model may be insufficient for precise prediction of individual pollution sources or plastic types. Furthermore, the prediction model should work even in the presence of spectral variations due to, e.g., varying hardware components or acquisition parameters in measurement setups, or chemical additives in the sample. This shows that we need mathematical methods that scan through the high-dimensional spectral data to discover common spectral features suitable for robust sample identification.

One field of such mathematical analysis is supervised machine learning (ML). Starting with the input data, given, e.g., by a set of spectra of representative samples, these methods generate models to classify the samples into their prescribed known categories like plastic types. Predictions of such models are based on learned combinations of spectral features. However, unprocessed raw features such as intensities at all wavelengths are high-dimensional and there are many possible parameters to fit the relatively limited information in form of prescribed sample classes. Such a situation often results in models that correctly predict sample properties for the training samples, but fail to generalize leading to low prediction accuracy for newly measured plastic samples.

To improve model generalizability, we reduce the dimensionality of the input data. This dimensional reduction (DR) process should retain all essential information in the raw data^36,37^. It plays a central role in identifying predictive spectral features. This process is unsupervised and thus, only the raw spectra are used as input and no additional information such as plastic type is used. Instead, a mathematical model is employed. Here, we utilize a recently published method termed Signal Dissection by Correlation Maximization (SDCM). SDCM has successfully revealed and dissected overlapping activating and inhibitory signatures in complex gene expression data from many patients in molecular oncology^38^. The spectral data from many samples are similarly complex as they measure the net effect of many unknown (but potentially plastic-specific) sources with stimulating (activating) or absorbing (inhibitory) influence on the measured light emission. Therefore, we believe that SDCM is able to extract information on the sample’s origins from the input spectra.

ML approaches have already been used to analyze spectral data in a variety of contexts. Most studies use supervised methods. For example, Li et al. used neural networks to distinguish THz data of metal and non-metal materials^39^. Liu et. al combined dimensional reduction with support vector machine (SVM) to classify spectral data of breast invasive ductal carcinoma^40^. Huang et al. have used principal component analysis (PCA) in combination with a regression model to classify mouse liver injuries characterized by THz spectra^41^. In material science, the spectral identification of components has been realized with a support vector regression (SVR) model^42^ and supervised classification scheme has been developed for laser-induced breakdown spectroscopy data on polymers and plastic^43^. In astrophysics the analysis of multifrequency data by supervised methods has been used for the classification of blazars^44^ and stars and galaxies^45^. Unsupervised approaches to spectral data are rare, but a few examples are the identification of preflare spectroscopic signatures^46^ and the mapping the diversity of galaxy spectra^47^.

Successful ML applications generally require vast amounts of data for learning, which explains why ML for plastic identification has so far primarily been used with Fourier-transform infrared spectroscopy^48–50^. As PL spectroscopy has the potential to easily produce even more high-throughput data, the combination with ML models seems very promising. However, such applications so far remain unexplored. Our study aims to fill this gap. Our ML approach for PL-based identification of plastic litter puts particular focus on the capabilities of ML models to discover spectral features that enable robust prediction of identifying sample properties, such as sample color, for newly collected plastic litter. It would be particularly useful to implement a classification method utilizing unsupervised DR as such an approach is more flexible and robust in dealing with new data. Here, we use such a method (SDCM) for dimensional reduction that we combine with a selection of commonly used supervised classification methods. Our results demonstrate that most supervised ML algorithms are able to predict plastic litter characteristics based on PL, underlining the physical information contained in these spectra. SDCM stands out as it helps models to identify spectral features that are specific to a single sample. Such specificity might allow us to adjust future plastic litter mitigation strategies to particular pollution sources more effectively.

## Results

Plastic litter is frequently associated with marine pollution. Therefore, we chose to evaluate the suitability of ML for PL-based identification of samples in the marine environment. For this purpose, we use a set of pristine plastics, plastics derived from consumer products and marine organic samples. All spectra were measured with the PL setup described in the methods section.

### Variations in the PL spectrum between samples

**Figure 1:**
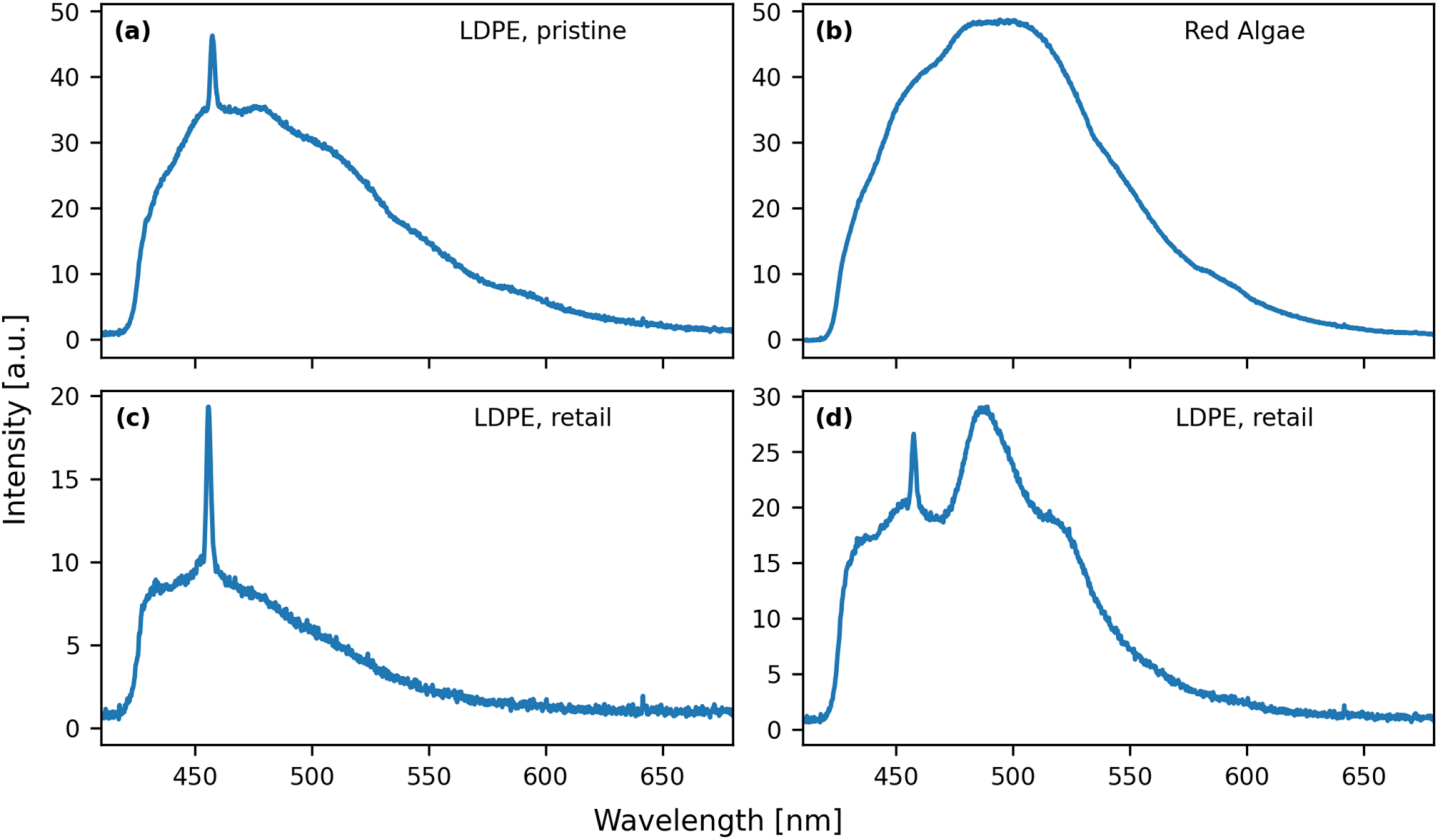
A representative selection of PL spectra of samples used in this study. The spectra correspond to (**a**): pristine LDPE, (**b**): red algae and (**c**,**d**): a consumer product made of LDPE. The spectra in (**c**,**d**) correspond to the same sample but were acquired with different alignments of the setup components, laser power values, and exposure times.

A PL spectrum of a sample describes the intensity of the emitted light at different wavelengths. To give an example, we show in Fig. 1 the spectra of three representative samples, namely **(a)**: pristine low-density polyethylene (LDPE), **(b)**: red algae and **(c)**, **(d)**: a consumer product made of LDPE. The plots illustrate the dependency between the spectral shape and the sample, which potentially allow us to identify samples with PL spectroscopy. Fig. 1c,d present the spectrum of the same sample acquired with different alignments of the optical components in the setup. We include these spectra in our library to account for measurements where the alignment in our setup is not optimal. In all spectra for LDPE, i.e. Fig. 1a,c,d, we also observe a Raman peak at around 450 nm. It must be noted, that additional spectral variations can also arise due to the inhomogeneity of the sample. We accounted for these variations by taking measurements at different sample sites (see Methods). In practice, the occurrences of additional peaks and spectral variations are unavoidable because of the lack of standards for measuring plastic samples and for building a PL setup. Combined with the diversity of samples from the marine environment, these variations imply that spectral libraries are always incomplete as it is impractical to capture all spectral variations. However, with respect to ML models complete libraries may not be necessary if the model can derive generalized selection criteria based on spectral features in the limited library. These features will be evaluated later in this study.

### Overall performance of ML models

In the following, we briefly summarize our procedure of constructing ML models and evaluating the data. The data analyzed consists of 1294 measurements from three different sample categories and 23 material types, which we combined to nine classification categories, for details see Methods and Table 3. All ML models designed here take as input the intensity data from PL spectroscopy, and yield a predicted material type. A detailed description of the implementation of DR, ML model generation, data preparation (see Fig. 8) and classification (see Fig. 10) can be found in the Methods section. A ML model can be generated with any combination of DR method and classifier. Its prediction performance depends on that combination and the intended application. To show the applicability of using PL spectroscopy data for ML, we demonstrate our results for five commonly used classifiers and two DR models which are summarized in Table 1.

**Table 1:** Overview of selected classifiers and DR methods used for the generation of ML models.

	Abbreviation	Name
Classifiers	GNB	Gaussian Naive Bayes^51,52^
	SVM	Support Vector Machine^51,52^
	Nu-SVM	Nu-Support Vector Machine^51,53^
	LR	Logistic Regression^51,52^
	RF	Random Forest^51,54^
DR methods	PCA	Principal Component Analysis^55,56^
	SDCM	Signal Dissection by Correlation Maximization^38^

We analyze the prediction performance of each ML model, by calculating three quantities, namely accuracy, precision and, recall (see ’Sample classification’ in Methods). These quantities can take values between 0% and 100% and are calculated with respect to a single property, e.g. the color green. The accuracy describes the fraction of correct predictions made by the model. A value of 100% implies that all predictions are correct. Considering each material type individually, we compare model predictions with the true material types, by calculating the precision and recall combined as $f_1$ score (see definition in Methods). The precision describes the fraction of positive predictions that are correct, while the recall gives us the fraction of actual positives that were correctly identified. A $f_1$ value of 100% implies that the model achieved the highest precision and recall. The $f_1$ score is then calculated for each material type individually and then averaged. In the following, we only focus on the accuracy and the $f_1$ score.

The ML model generation starts with a preparation of the spectral data to ensure that all spectra are treated equally. The steps involved for this preparation are illustrated in Fig. 8. Our workflow to create the ML models is presented in Fig. 10 which consists of three commonly used consecutive stages: training, testing, and validation, see Fig. 9. For each stage, we use an individual set of spectral data. The first two stages are used to optimize the model parameters for the prediction. In the last stage, we test the optimized model on a data set unknown to the model to calculate the prediction metrics described earlier. We then use these results to benchmark the performance of our ML models.

**Figure 2:**
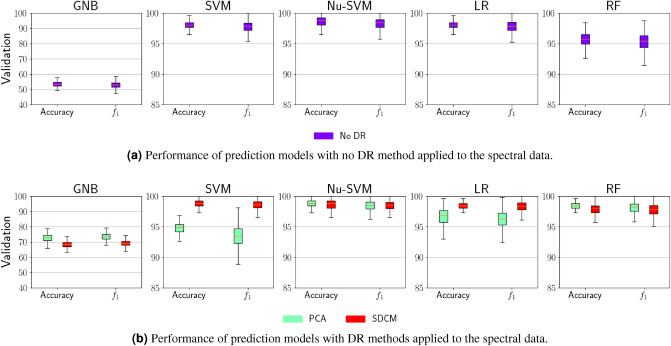
(**a**) Performance of prediction models with no DR method applied to the spectral data. (**b**) Performance of prediction models with DR methods applied to the spectral data. Overview of the prediction performance of ML models for PL-based sample identification. The accuracy and the $f_1$ score are presented as box plots and are calculated during the validation stage. The colored boxes show the quartiles of the achieved scores, while the whiskers extend to show the rest of the distribution. Each plot presents the prediction performance with a different classifier. All plots in (**a**) show the performance when the spectral data has not been processed with a DR method. All models in the plots in (**b**) are built around spectral data that have been processed with either PCA or SDCM. The prediction performance at all training stages are summarized in Fig. S1.

We first evaluate the performance of those ML models that use the unprocessed spectral data set as input. This will later allow us to analyze the benefits of applying DR methods on the spectral data set for PL-based plastic litter identification. Figure 2a summarizes the performance for all five models. Each plot shows the model performance for one classifier. In a single box plot, we find the calculated accuracy and $f_1$ score. We clearly see that most models achieve values of over 90% for both quantities. The model that is built with Nu-SVM stands out as it achieved the highest performance values. However, models that use the classifier GNB perform significantly worse with an accuracy and a $f_1$ score are around 55%. Figure 2b shows the prediction performance of the models using data preprocessed with a DR method as input, i.e. SDCM or PCA. We observe that all SDCM-based models have an improved performance compared to the models in Fig. 2a. Here, the classifier GNB benefits the most from SDCM as the accuracy and the $f_1$ score of the corresponding model increase to roughly 70%. As for the remaining models, we see a slight increase by at most 2%. For PCA-based models, we find an improved performance comparable to SDCM-based models when it is combined with the classifier GNB, Nu-SVM or LR but a performance drop by up to 3% when we use the classifier SVM or LR. Interestingly, we see a trend that linear classifiers (SVM, Nu-SVM and LR) work better with SDCM-transformed data. This could be relevant for creating interpretable classification models with SDCM, as linear classifiers can be easier understood in terms of their classification rules than the non-linear ones.

Our evaluation reveals a high prediction performance for most of the ML models generated. We see that the choice of the classifier has the largest influence on the model’s performance. Furthermore, preprocessing the spectral data with a DR method does not always lead to prediction improvements so that comparisons between the models are required to identify the optimal DR method and classifier combination. Note that additional improvements might also be achievable by optimizing the parameters bound to the classifiers.

### Prediction performance on sample types

Bad drops of a model might be traced back to specific sample types in our set. For example, a prediction model may specifically struggle to distinguish between samples made from PET and PS. This would then cause a drop in the overall performance even though the prediction for the remaining samples is high. To test if such cases occur for our models, we now evaluate the prediction performances for individual sample types.

**Figure 3:**
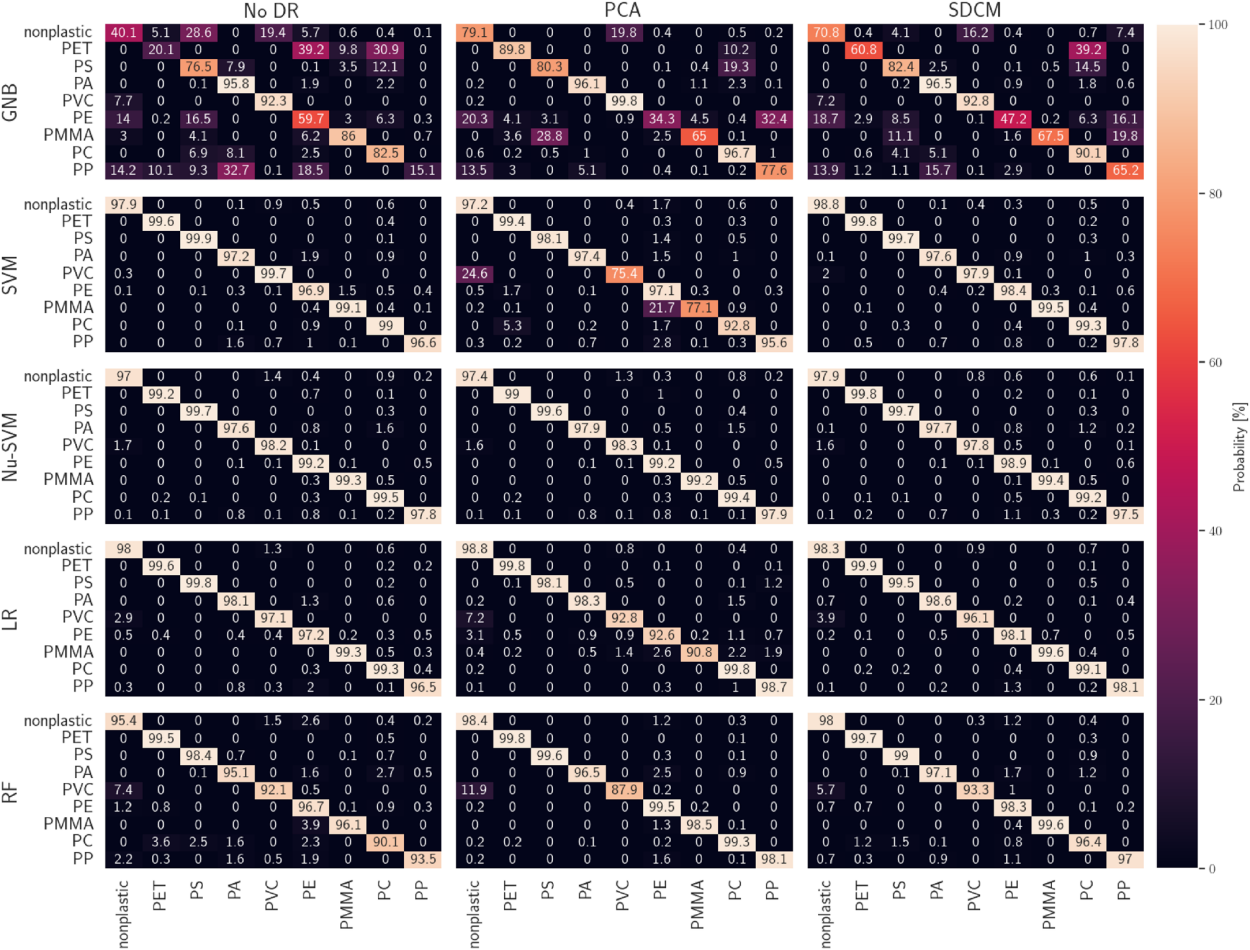
Confusion matrices of the validation set for individual sample types. Each matrix corresponds to a model generated with a unique classifier and DR method combination. A matrix is to be read as the probability (in %) that the sample type specified in a row is classified as the type specified in the column.

We conduct this analysis by evaluating the confusion matrix of the validation data set for each ML model. In the field of machine learning confusion matrices are the standard way of presenting the performance of a learning algorithm. Rows of the matrix represent the actual class while columns represent the predicted class. The calculated confusion matrices are presented in Fig. 3. The entries of each matrix are the probability (in %) that the sample type specified in a row is classified as the type specified in the column. For example, for the model generated by applying GNB on the entire spectral data (top row, first column in Fig. 3), the probability that a non-plastic material is identified as PS is 28.6%. By definition, all values in a single row add up to 100%. In case of a perfect prediction model, all entries along the main diagonal are 100% while the off-diagonal ones are 0%. All matrices in the first column of Fig. 3 correspond to models trained with unprocessed data. Here, we observe that for all models, except those that use the GNB classifier, a high identification accuracy of over 95% for all sample types. For example, the LR-based model achieves an accuracy of 98% for non-plastic samples. We observe no sample types where the identification could be difficult for the model. Further improvements for all models can be achieved by preprocessing the data with SDCM where the probability for a correct prediction per sample type is on average 3% higher. For models that use PCA processed data as input, we also observe an improvement with an average prediction performance per sample type of 2.2%. We find a slight increase for Nu-SVM-based models while SVM-based models appear to struggle to predict PVC and PMMA correctly. This worse performance agrees with our findings above and emphasizes once more that the models must be compared with each other to find the optimal classifier and DR method combination.

### Detecting identifying characteristics in PL spectra

Our previous results demonstrate that with ML models it is possible to classify with high accuracy PL spectra into their respective plastic types. Besides classification, we are also interested in finding features in the spectra which are characteristic of the various sample properties such as type, color, or manufacturer. The question whether such spectral fingerprints exist is relevant for microplastic detection, as they allow us to efficiently pin down their sources. Furthermore, they may be analyzed to determine the underlying chemical structure.

At the *no DR* training stage of the previous section, the classifier sets up geometric borders in the input space of wavelength-bins to distinguish the spectral regions that are characteristic for different sample types. Reshaping these complex regions into an understandable spectral representation to use as fingerprints is a difficult task. For supervised classifiers this can be achieved by using, e.g., convolutional neural networks^57^. However, as mentioned, our goal is to establish an unsupervised approach for which we need to develop our own interpretation scheme.

To do so, we make use of the fact that measurements of samples that have the same properties, for example a specific color or any physical, chemical, or systemic property, should have common features in their spectra. These features are not necessarily a set of peaks in the spectra but they can also be a complex relationship between the intensities at different wavelengths. This operation removes all irrelevant information from a measurement, which makes it easier to identify collections of measurements with similar properties. These collections are here referred to as *clusters*.

In the following, we discuss the relationship between clusters detected by the DR methods and the sample properties. We introduce a metric that quantifies this relationship and a method of deriving quantifiable associations between clusters and properties.

#### Labels and metrics

We divide the sample properties of a measurement into the following categories: $<<is plastic?>>$ (whether sample is a plastic or not), $<<origin>>$ (either manufacturer, nature or retail), $<<color>>$ (sample color), $<<type>>$ (material type) and $<<sample ID>>$ . The latter is a unique identifier for each plastic sample probed. As the samples have been probed multiple times, the replicates of each sample may carry information that is specific to the sample (e.g. due to specific chemical composition). Such individual characteristics might be useful in applications where the task is to backtrace the origin of a particular production source of plastic. All categories are discrete and finite valued. We refer to a set of values from one or more categories as a label.

As mentioned earlier, the two DR methods we use in this study are PCA and SDCM. PCA is a conventional method, which separates the input data into linear clusters along orthogonal axes that maximizes the variance along each axis. SDCM is a novel method, which separates the data into monotonic clusters along non-orthogonal axes, maximizing the local correlation. If a spectrum belongs to a cluster can be calculated from a quantity called cluster weights. Using this quantity, we say a measurement belongs to a cluster, if its weight is above a given threshold. While SDCM produces these weights as part of its output, they have to be estimated from the clustering coefficients for PCA. For PCA, the choice of threshold also is more critical than in SDCM (see supplementary information for details).

If there is a relation between a cluster and a label, then most of the measurements belonging to that cluster should also carry that label and vice versa. Therefore, it is sufficient to determine the agreement between the list of measurements belonging to a cluster and the list of measurements that carry that label. A good metric quantifying this agreement is the $f_1$ score, which is also known as *F*-score. It is defined as the harmonic mean between *precision* and *recall*, which quantify the error rate of false and missed associations, respectively. If the number of measurements for a label is large relative to the size of the data set, a high $f_1$ score may be achieved by random association. Such cases can be filtered by calculating the probability *p* of random association with a hypergeometric test. We only consider those $f_1$ scores with $p\le 0.005$.

#### Associating one cluster to one label

We test the association of a cluster to a label by calculating the $f_1$ score for every possible combination of cluster and label. Labels can be drawn from one or more categories, e.g. “PVC” drawn from $<<type>>$ or “PVC, red” from the combination $<<type, color>>$. In a sample set of limited size it is unlikely that every theoretically possible label is represented, due to the fast growth of the number of possible combinations of each category. In consequence, many labels are often related. For example, if all samples of type “PVC” have the color “red”, and all “red” samples are of type “PVC”, then the labels “PVC”, “red” and “PVC, red” are equivalent descriptions of the underlying set of measurements. If a cluster has a high $f_1$ score for these measurements, any of these three labels could provide an interpretation, and choosing a specific one requires an additional and arbitrary selection rule. We therefore group together all labels which belong to the same set of measurements into an “equivalence class” (EC). Each EC is associated to one (unambiguous) set of measurements and to one or more equivalent label descriptions. To evaluate the performance of the DR methods, it is sufficient to test every cluster against the set of measurements in each EC. Our data set provides 141 different ECs.

**Figure 4:**
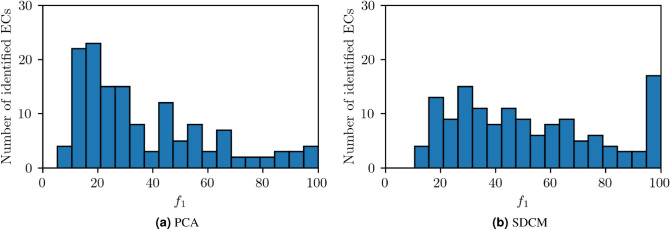
(**a**) PCA, (**b**) SDCM. The histograms show the distribution of $f_1$ scores of each match for PCA and SDCM. A large $f_1$ score corresponds to a close match between cluster and EC.

Figure 4 shows the number of ECs matched to a cluster for different $f_1$ ranges. The results are presented for the clusters found in the data preprocessed with PCA (Fig. 4a) and SDCM (Fig. 4b). One sees that several matches at high $f_1$ scores are found. SDCM finds a total of 11 *perfect* matches with $f_1 = 100$, whereas PCA finds one. This shows that both DR methods are capable of finding several clusters which can be associated to a specific sample property.

As most ECs are associated to multiple potential labels, the “correct” label description is not readily available. Among all 141 ECs, we find 4 that are associated with a single label (see Table 2 for a listing and their $f_1$ values). To obtain a meaningful interpretation of the remaining ECs, we evaluate them with two different rules for selecting a preferred label. For example, if a cluster matches with an EC which contains the three equivalent labels “PVC, red, retail”, “PVC, red” and “PVC”, then all PVC samples have red color and were drawn from retail products. We can

(A) always select the label which represents the *smallest* number of categories (i.e. “PVC”), or
(B) always select the label which represents the *largest* number of categories (i.e. “PVC, red, retail”).

In case no preferred label can be selected the match is dismissed. (A) always looks for the simplest and (B) to the most specific possible description. Rule (A) this comes with the potential risk of overestimating the generality of the description, while rule (B) comes with the risk of underestimating it. Of course, other selection rules are just as valid. In particular an expert could analyze the data by hand and decide for the most suitable label.

**Table 2:** $f_1$ scores of the four ECs which only contain a single, unambiguous label.

Category	Label	PCA	SDCM
Is plastic?	Plastic	39.4	71.4
Color	White	31.5	53.5
Color	Yellow	55.0	64.5
Is plastic?, color	Plastic, white	29.0	51.0

**Figure 5:**
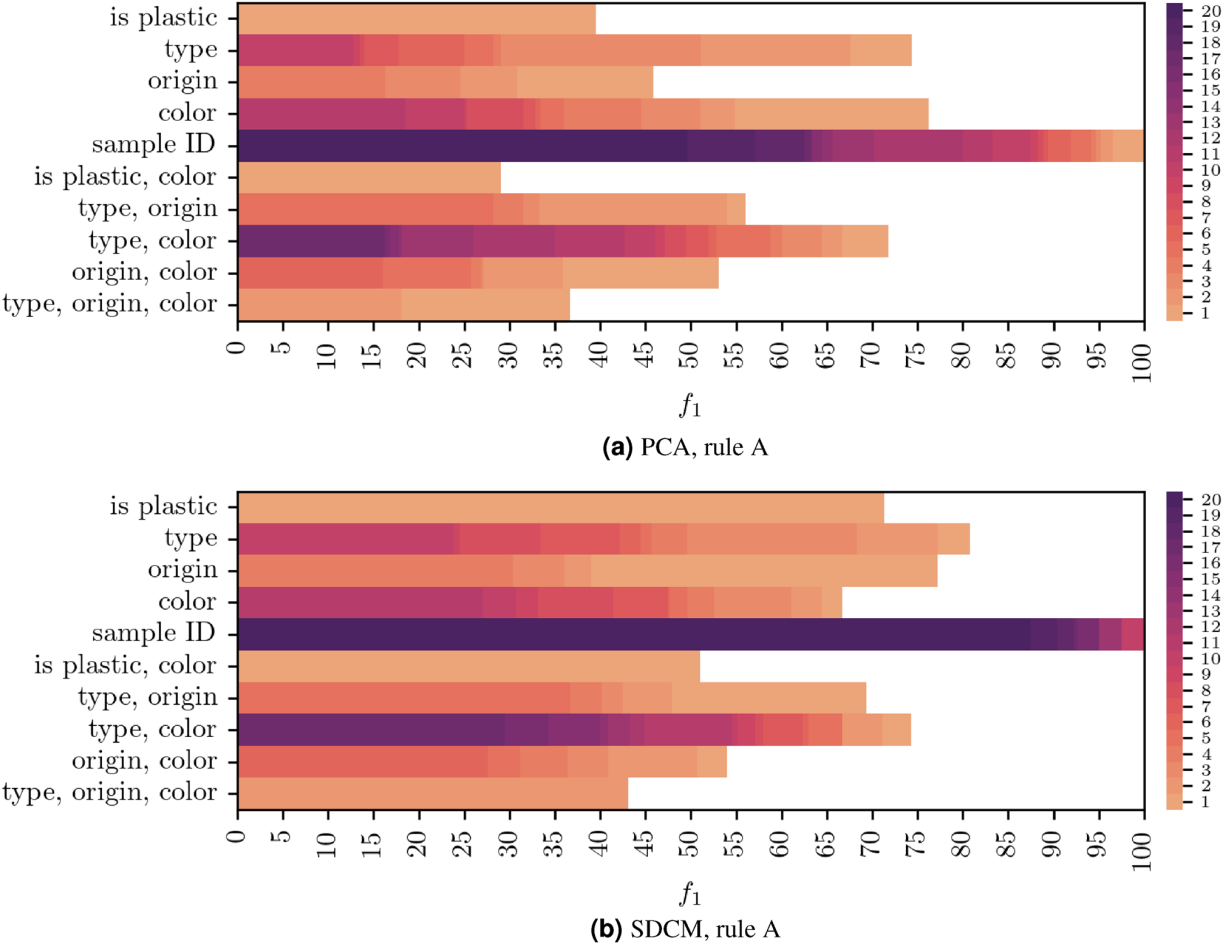
(**a**) PCA, rule A. (**b**) SDCM, rule A. Cumulative distributions of matched labels over $f_1$ in the categories defined by rule A. For each set of categories, which are defined by rule A, the figures show the number of labels that can be matched with at least one cluster at a given $f_1$ score or higher. The color scale is capped at 20 to improve readability.

**Figure 6:**
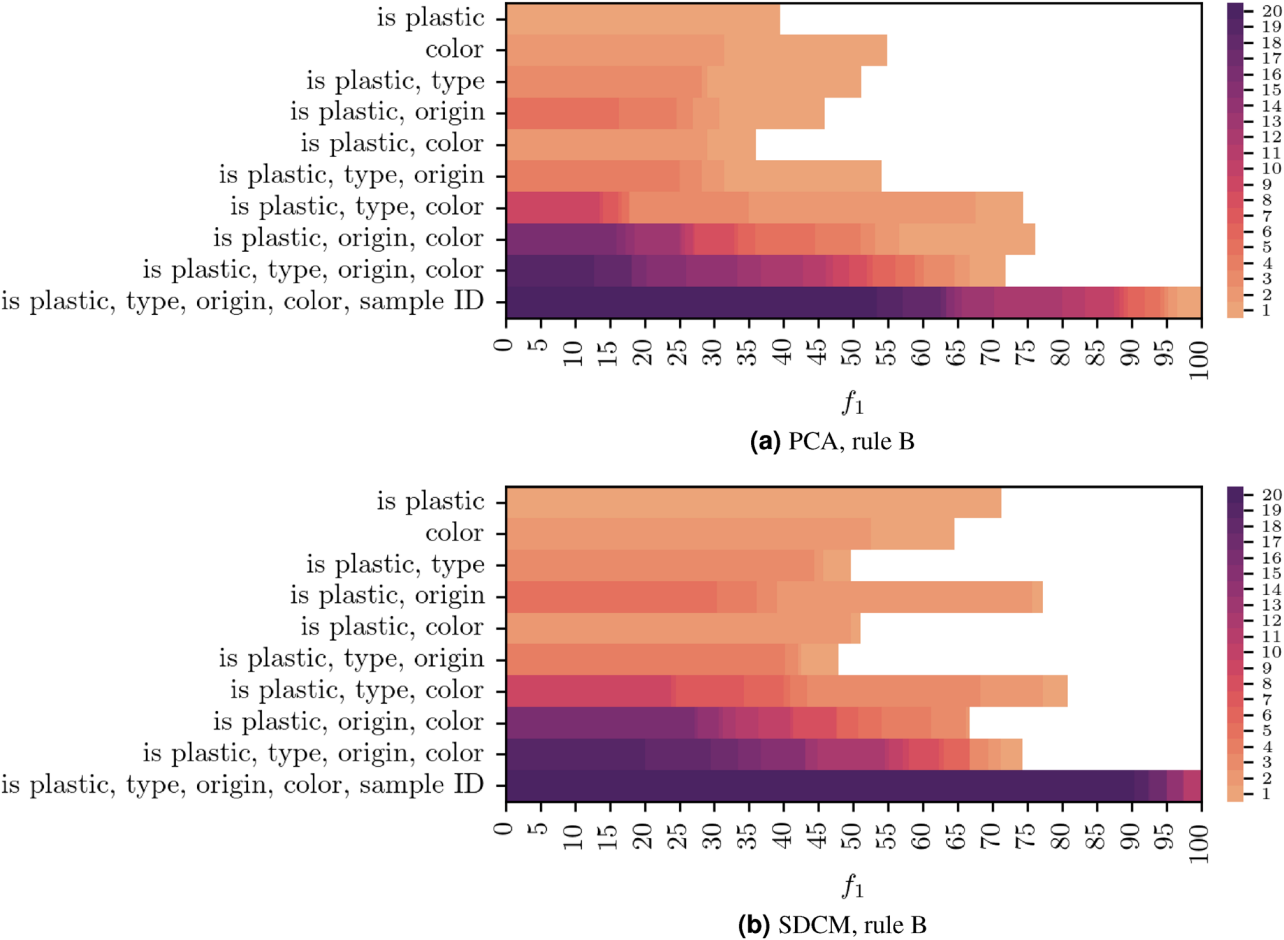
(**a**) PCA, rule B. (**b**) SDCM, rule B. Cumulative distributions of matched labels over $f_1$ in the categories defined by rule B. For each set of categories, which are defined by rule B, the figures show the number of labels that can be matched with at least one cluster at a given $f_1$ score or higher. The color scale is capped at 20 to improve readability.

We apply these rules on the data processed with PCA and SDCM. The results are summarized in Figs. 5 and 6. For each set of categories, which are defined by the selection rules, the figures show the number of labels which can be matched with at least one cluster at a given $f_1$ score or higher. For general sample properties, such as $<<type>>$ or $<<color>>$, both figures reveal matches with $f_1$ values that are at most in mid-range. Thus, broad physical characteristics do not seem to be resolved as single, distinct spectral features that appear in a single cluster. In some cases, these characteristics might not be represented in the measured PL spectra at all. For example, the production process might not have an effect on the PL spectra such that there is no spectral fingerprint for different manufacturers. The data may also be more heterogeneous than the labeling indicates, in this way giving rise to property sub-types with several distinct fingerprints, forming separate clusters.

On the one hand, we previously observed that ML models can accurately identify sample types from their spectral data. On the other hand, we found that no general sample property, such as type, manufacturer or color, can be associated to a single cluster with a high $f_1$ score. Such properties therefore are not represented as distinct spectral fingerprints of various fixed peak intensities in the input data. Rather, their spectral representation may depend on other sample properties, which may not only induce changes in peak intensities, but also overall shifts along the spectrum. Due to this complex behavior, high-dimensional machine learning is required to identify the sample type and other properties.

We find high $f_1$ values, i.e. $f_1 > 90$, for those labels that contain the category $<<Sample ID>>$, which enumerate the samples from which the spectra were recorded. This means that characteristic spectral shapes of individual samples are sufficiently fixed in their intensities across all measurements that can be resolved with both DR methods. In the data preprocessed with PCA, we find one perfect match with $f_1=100$ value for both rules. In comparison, SDCM performs significantly better. Here we find 10 perfect matches with rule A and 11 perfect matches with rule B. Our findings imply that while both DR methods can detect sample specific spectral features in the data, SDCM is more effective in finding these features. As a result, SDCM is particularly useful for identifying characteristics that allow particle tracking to a single source.

Finally, we would like to point out that further additions of diverse measurements might reduce the ambiguity in choosing a representative from the ECs and improve the detection of clusters which can be associated to more general physical properties.

## Discussion

Significant progress in the field of microplastic research will only be achievable if we obtain a better quantitative understanding of the current status quo. This requires to perform worldwide measurement on plastic litter distributions and compositions. This is challenging from both an experimental and theoretical perspective. Experimentally, samples have to be characterized according to standard protocols ideally with simple setups that could be available worldwide. Theoretical analysis approaches then have to be able to extract from vast amounts of experimental data the relevant material parameters.

In this study we have demonstrated that such a combined approach could rely on photoluminescence spectroscopy that is being analyzed by our machine learning based theoretical approach. To do so, we evaluated the capability of ML models to identify plastic and non-plastic materials based on their PL spectra. Our results reveal that most of the ML models can achieve high prediction performances with accuracies over 95%. In particular, the models that were combined with SDCM achieved the highest performance.

Furthermore, we tried to identify potential links between the characteristics in the sample spectrum and the sample properties. Such links could lead to selection criteria that apply to plastics in general. For this purpose, we analyzed the data that have been processed with PCA and SDCM. In our analysis, we found that the SDCM algorithm particularly stood out in finding criteria that apply to specific samples. Our result could proof to be particularly useful for environmental studies. For example, they can provide means to identify local plastic litter sources and thus, can help to create more effective plastic litter reduction policies.

Our approach could provide the first step for analyses performed on large scales. As the best preforming combination is based on an unsupervised learning technique, we expect our approach to be robust against alternation of the input data, i.e. to perform similarly well for new data that significantly differs from the currently available spectroscopic data. Of course, this has to be tested for example by the establishment and maintenance of a spectral library with complete sample records and additional experimental heterogeneities. To do so, it is necessary to define guidelines which assure that each record in the library is complete and accurate.

Such a library would allow further tests to evaluate the extent and reliability of our method. In particular, we could then evaluate the performance of our ML models for new measurements that are not present in the library.

## Methods

### Experimental setup

**Figure 7:**
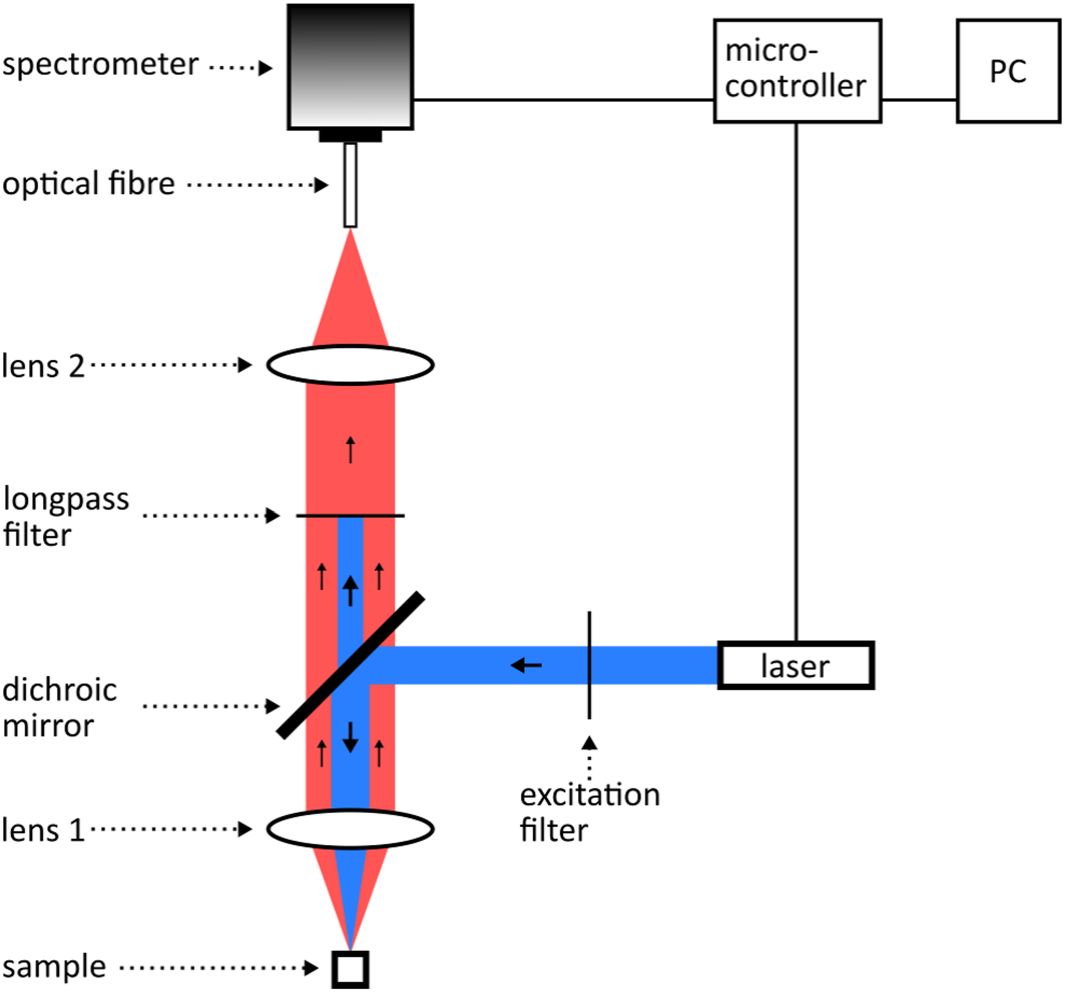
Illustration of our PL spectroscopy setup. The excitation light follows the path highlighted in blue to induce PL on the sample. The pathway of the PL signal is highlighted in red.

Figure 7 illustrates our experimental setup for PL spectroscopy measurements. The blue path highlights the incident beam which excited the sample and induced photoluminescence. The central wavelength of our laser (SF-AW210 with TTL driver, InsaneWare) depends on the laser power and varied between 402 nm and 404 nm. To narrow down the excitation bandwidth, the generated light passed through an excitation filter with a central wavelength and a bandwidth of 405 nm and 10 nm, respectively. A dichroic mirror directed the light to lens 1 which focused incident light on the sample’s surface. The path taken by the emitted photoluminescence light is highlighted in red. Starting from the sample’s surface, this light was collected and collimated by lens 1 and passed through the dichroic mirror. To ensure that the excitation light was completely removed from the emission path, we used a long-pass filter with a cut-on wavelength of 420 nm. Finally, lens 2 focused the light onto an optical fiber, which directed the light to our spectrometer (LR2, Lasertack GmbH).

Both the laser and the spectrometer were controlled with a microcontroller (Mega 2560, Arduino) which, in turn, was connected to a computer. This arrangement made it possible to control the laser power, exposure time, and the time between sample excitation and signal acquisition. The latter was set to 500 ms.

### Samples and measurement parameters

Samples from the environment show a large diversity since interactions with the environment can alternate the chemical composition. Therefore, spectral libraries can always be considered as unbalanced and incomplete as it is impossible to reflect the sample variety in a single data set. To account for these conditions in our study, we generated our spectral data set from 46 samples, which consisted of non-plastic materials from the marine environment and plastics from different manufacturers and retail products. A summary of the data set is presented in Table 3. For each sample, we adjusted the laser power $\mathrm {P_{laser}}$ and the exposure time $\mathrm {t_{ex}}$ to acquire a signal with a low background noise. A list of these measurement parameters is given in Table 4. To introduce additional inhomogeneities in the spectral library, we included an additional measurement for eight samples, where we readjusted the alignment of the optical components. For these samples, the two sets of spectra represent variations when the components are aligned or not. All spectra were measured over the full range of the spectrometer, i.e. between 200 nm and 1000 nm. For each sample and setup, we took 9 to 20 measurements to capture spectral variations due to sample inhomogeneity. In total, all samples were measured 29 times, with the exception of four non-plastic samples, which were measured 19 times. We also took measurements of the background noise, which was required in our ML model building process.

**Table 3:** Overview of samples used for this study.

Sample category	No. of samples	No. of measurements	Material type
Non-plastic	12	308	Sand
			Wood
			*Posidonia oceanica* (plant)
			*Sepia officinalis* (bone)
			*Echinocardium cordatum* (shell)
			*Hexaplex eggs* (shell)
			*Monodonta turbinata* (shell)
			*Neverita josephina* (shell)
			*Lithophyllum racemus* (algae)
Plastic (manufacturer)	26	754	Polyamide (PA)
			Polycarbonate (PC)
			Polyethylene (PE)
			Low-density polyethylene (LDPE)
			High-density polyethylene (HDPE)
			Polyethylene terephthalate (PET)
			Polymethylmethacrylate (PMMA)
			Polypropylene (PP)
			Polystyrene (PS)
			Polyvinyl chloride (PVC)
Plastic (retail)	8	232	LDPE
			HDPE
			PET
			PP

**Table 4:** Summary of samples and measurement parameters. For each sample, the laser power ($\mathrm {P_{laser}}$), the exposure time ($\mathrm {t_{ex}}$) were adjusted.

Sample category	measurement	
	$\mathrm {P_{laser}}$ [mW]	$\mathrm {t_{ex}}$ [ms]
Non-plastic	0.2, 0.5, 2.5, 2.6, 2.8	300
Plastic (manufacturer)	0.5, 2.5, 5, 20, 25, 30, 50, 100	300
Plastic (retail)	0.5, 5, 25, 50, 104, 130	300, 1500

### Dimensional reduction and SDCM

Dimensional reduction (DR) aims to project high-dimensional data, e.g. spectra measured over a large number of wavelength bins, onto a lower-dimensional space. In this work, we used both a conventional method called Principal Component Analysis (PCA) and a novel method called Signal Dissection by Correlation Maximization (SDCM) to achieve a DR in our data.

SDCM is an unsupervised algorithm for detection of superposed correlations in high-dimensional data sets^38^. Conceptually, it can be thought of as an extension of PCA for non-orthogonal axes of correlation, where instead of projecting out detected dimensions, the discovered axes of correlation are iteratively subtracted (*dissected*) from the data. Initially developed for the application in bioinformatics for the clustering of gene expression data, it can be generically applied on any high-dimensional data containing (overlapping) subspaces of correlated measurements.

We denote by ${\mathbb {M}}^{N_f,N_m}$ the set of real valued $N_f \times N_m$ matrices, where $N_f$ is the number of features in the data and $N_m$ the number of measurements. The $N_f$ row vectors and the $N_m$ column vectors belong to different vector spaces referred to as *feature space* and *measurement space*, respectively.

The main assumption of SDCM is that the input data, ${\mathcal {D}} \in {\mathbb {M}}^{N_f, N_m}$, is a superposition ${\mathcal {D}} = \sum _{k=1}^n E_k + \eta$ of submatrices $E_k \in {\mathbb {M}}^{N_f, N_n}$ (also called *signatures*) and residual noise $\eta$. We interpret $E_k$ as a physically meaningful hypothesis in the data, e.g. a common physical or chemical property, due to which some samples and features are correlated. As superposing is a non-bijective operation, we need further conditions to dissect ${\mathcal {D}}$ into separate $E_k$. We assume that each $E_k$ is *bimonotonic*, i.e. that there exists an ordering $I_f$ of the $N_f$ indices and an ordering $I_m$ of the $N_m$ indices such that the reordered matrix $\tilde{E}_k = E_k(I_f, I_m)$ is monotonic along all rows and columns. Thus, after reordering, the correlations follow monotonic curves in both feature- and measurement space. While this bimonotonic requirement restricts the applicability of the algorithm, it allows an unambiguous dissection of ${\mathcal {D}}$ into the $E_k$ components. In contrast to PCA, it also allows detection of non-linear (bi)monotonic correlations, whose axes are non-orthogonal.

SDCM dissects the data in four steps:

1. Detection of initial representatives for an axis of correlation. in both feature and measurement space.
2. Calculation of the signature axes by maximizing the correlation.
3. Estimation of the bimonotonic, possibly non-linear, correlation curves (*eigensignal*) in both feature and measurement space. For this purpose, a non-parametric regression is used.
4. Subtraction of the data points belonging to the eigensignal from the data set.

These four steps are performed iteratively until no more representatives of axes can be found. SDCM treats rows and columns completely symmetrically. Each feature and sample is given a strength value *s* and a weight value *w* for every signature. The strength value (in units of the input data) quantifies the position along the eigensignal. The weight $w\in [-1,1]$ quantifies how strongly the feature or the sample participates in the signature, i.e. how close to the eigensignal it is. Typically, the number of signatures detected will be orders of magnitudes smaller than the number of input features, and in this way give rise to an effective DR of the data.

### ML model generation

To generate our ML models for PL-based sample identifications, we chose a combination of supervised and unsupervised learning methods. In the following sections, we describe all steps used to generate these models.

#### Data format

We saved the information of a spectrum in two different files: one file that contains the absolute intensity as a function of wavelength; and one that contains details about the sample and the measurement. The latter provides labels for all spectra, which are central for the evaluation of the classifier’s performance. We use the following categories:

- *Type*: material type of the sample.
- *Origin*: name of the manufacturer or location. All retail samples have the same label.
- *Color*: color of the sample.
- *is plastic*: specifies if the sample material is a plastic or not.
- *Sample ID*: unique ID identifying the sample from which multiple replicate measurements have been taken.

All categories are discrete and finite valued. In the following, *i* enumerates the set of features (spectral bins) $f_i \in \mathcal {F}$, $N_f := |\mathcal {F}|$ and *j* enumerates the set of measurements $m^j \in \mathcal {M}$, $N_m := |\mathcal {M}|$.

#### Prediction categories

We aggregated the 19 distinct material types from Table 3 by combining all non-plastics into the type *nonplastic* and LDPE, HDPE and PE into the type *PE*.

#### Preparing the spectral data

**Figure 8:**
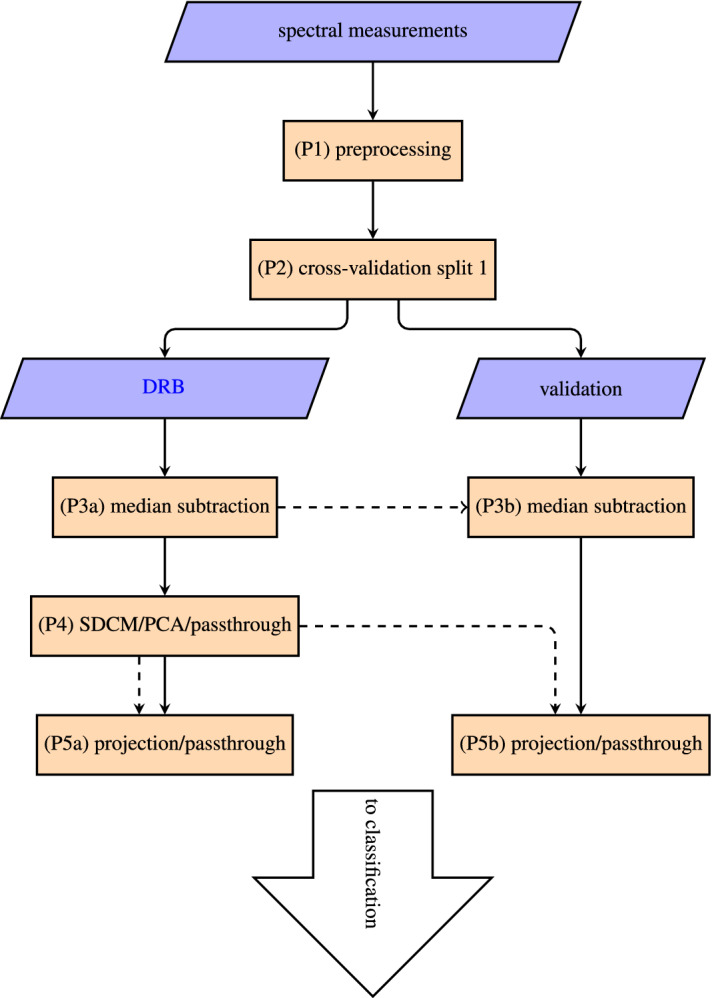
Flowchart of the data preparation pipeline. The solid arrows denote the data flow and the dashed arrows denote the influence by the parameters. The raw input data was preprocessed (P1) to remove background offsets and noise, to filter out overexposed measurements, to cut the data into the appropriate spectral range and to normalize it. The data was then split 25 times into 80–20% *DRB* and *validation* batches (P2). The median of each spectral bin was calculated across all DRB measurements and subtracted from both DRB and validation sets (P3a and P3b). The DR (SDCM, PCA) was applied to the DRB (P4) set. *Passthrough* denotes that no DR was applied for the *no DR* data set. The results were used to project DRB and validation into the dimensional reduced space (P5a and P5b). The final sets were used as input for the classification pipelines. Generated with pgf v3.1.9a.

In the following, we describe the data preparations applied to the spectral data before it is passed to the classification pipeline. The data preparation pipeline is explained in Fig. 8. The references (P1) to (P5) refer to the respective nodes in the flowchart.

To treat all spectra equally in the ML process, we needed to preprocess our data first (P1). We started by interpolating the spectral data and the corresponding background measurement onto a common spectral axis. The number of spectral bins was kept equal to the mean of the number of bins in the overall set. Then, we subtracted the background measurement from the sample spectrum. Once all spectra were processed in this way, we concatenated the data into a single matrix.

Since we did not expect any signal below the laser peak, we estimated the offset from the baseline $O^j$ for for the *j*-th measurement by calculating the median intensity in the range 294 nm 400 nm. Similarly, we estimated the noise level $\eta ^j$ by calculating the standard deviation in the same range. As we regarded any offset of the spectra as systematic, we subtracted it from the data.

To remove overexposed or noisy spectra, we applied a process that automatically filters out all data, which do not satisfy our conditions. We singled out measurements with experimental overexposure by determining for each spectrum the maximum $M^j$ of the smoothed spectrum of $m^j$. For the smoothing, we used a running median with a window size of 20 nm. The exposure level was then calculated as $E^j = \frac{O^j}{M^j}$. We then discarded overexposed measurements with $E^j < 0.5$. To detect noisy spectra, we calculated the signal-to-noise-ratio, $\textsf{SNR}^j$, with the expression

$$\begin{aligned} \textsf{SNR}^j = \frac{P^j}{\eta ^j}\text {.} \end{aligned}$$

Here $P^j$ is the power of the spectrum given by

$$\begin{aligned} P^j = \sqrt{\frac{1}{N_f}\sum _{j=1\ldots N_f} \bigg ({s}^i_j\bigg )^2}\text {,} \end{aligned}$$

and ${s}^i_j$ is the *i*-th spectral bin of $m^j$. We considered a measurement to be noisy if the signal-to-noise ratio is less than 2. Such measurements were then discarded.

To generate the model, we only considered the spectral information in the range 410 nm 680 nm, which contains most information about the sample. Each spectrum was then normalized such that the integral over its absolute values is one. This is particularly important for SDCM to ensure that the regression steps converge within a reasonable time.

#### Cross-validation splits

In our classification model, we split the data at the (unsupervised) DR stage and at the (supervised) classification stage.

**Figure 9:**
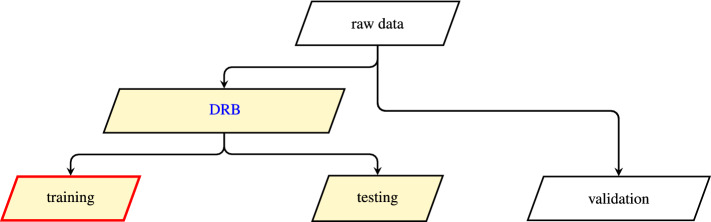
Conceptual description of data set splittings. The data was split before the DR process and once again before the classifier training. Yellow nodes took part in the DR process. The red border denotes that *training* was used to fit the classifier. Generated withpgf v3.1.9a.

In a real world application, the trained classifier pipeline is applied onto the novel data, which was not part of the DR or learning process. To properly assess our model’s performance, the data needs to be split into batches on which the model is trained, and batches on which its performance is evaluated. As SDCM is computationally expensive, we applied a two-step process in which the data was first split several times into multiple *dimensional reduction* batches (DRB) and *validation* batches with the DR method being applied to *DRB*. Each *DRB* batch was again split into multiple *training* and *testing* batches. The model was then trained on each *training* batch, and its performance was evaluated on the corresponding *testing* and *validation* batches. Figure 9 illustrates the conceptual differences in the different splits. This has the additional benefit of providing a comparison of the classifier’s performance on measurements, which have been part of the DR (*testing*) and novel measurements (*validation*). We note that there is no meaningful difference between *testing* and *training* if no DR method is applied.

The data was cross-validated 25 times with an 80%-20% split into a DRB and a *validation* set (P2). We used *type* and *sample ID* as stratification variables, i.e. we aimed to keep the relative proportions of each type and sample ID equal in *DRB* and *validation*. For stratification, we used the cvpartition methods of the MATLAB Statistics and Machine Learning toolbox (The MathWorks, Inc., Natick, Massachusetts, United States).

We calculated the median of each spectral bin in DRB and subtracted it from each measurement in both DRB and *validation* set (P3a and P3b). This centers the data at the zero level of DRB in each spectral bin. We additionally performed classifications of spectra and PCA without median subtraction. An evaluation showed that this process does not significantly influence the performance of the classifier. The final dimensions $N_f \times N_m$ of the DRB and the *validation* set for each cross-validation were $1394 \times 1036$ and $1394 \times 258$, respectively.

#### Dimensional reduction methods

In our study, we used SDCM and PCA as DR methods and compared them to the baseline when no DR was performed. The three input types are referred to as *SDCM* and *PCA* and *no DR*. SDCM and PCA reduce the data into signatures and principal components (PCs). From the processed data, we could derive strengths and PC coefficients for input into the classification pipeline.

We applied the DR methods on the DRB data (P4). For *no DR*, the data was just passed through. SDCM terminates with a median of 130 identified signatures over all cross-validation splits. PCA does not terminate on its own but rather produces a number of PCs equal to the number of measurements. To achieve an effective DR, we chose the first 130 PCs for further analysis.

Once SDCM finds a set of signatures in DRB, the strengths and weights relative to these signatures for *validation* needed to be determined. This is a non-trivial task, since the signature axes can be non-orthogonal and the method is dissecting and not projecting. The standard procedure is to repeat the dissection on the new data, while fixing the signature axes to the previously detected values. This, however, might still regress to a different eigensignal, which skews the prediction results. To circumvent this, we performed a weighted projection of the data onto the DRB signature axes (P5b), where the projection weights were the signature weights for each spectral bin calculated on DRB. This removes some of the precision obtained by SDCM, as spectral features explained by a single signature can still produce significant projection values in other signatures, if the axes are not sufficiently orthogonal. However, it ensures that all signature strengths are obtained relative to the same axes. For consistency, DRB is also projected onto the signature axes (P5a).

#### Sample classification

**Figure 10:**
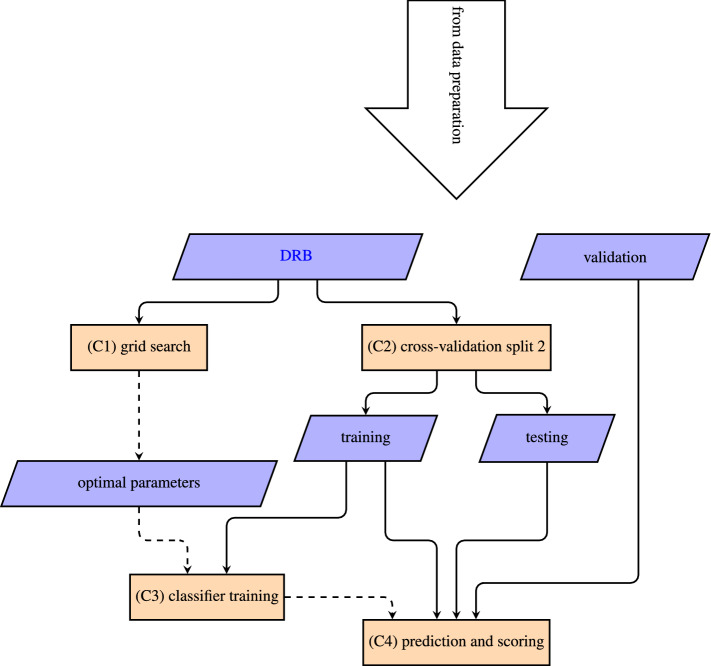
Flowchart of the classification pipeline. The solid arrows denote the data flow and the dashed arrows indicate influence of parameters. For clarity, the nodes for the data labels are omitted. DRB and *validation* were taken from each of the 25 validation splits made in the data preparation stage. For each classifier the DRB was used to optimize the classification parameters via a cross-validated parameter grid search (C1). DRB was then split 144 times into 80–20% *training* and *testing* batches (C2). The classifier was fit to *training* using the parameters found in the grid search (C3). Its performance was then evaluated over *training*, *testing* and *validation* (C4). Generated with pgf v3.1.9a.

Our classification pipeline consists of optimization of each classifier, followed by a cross-validated training and scoring. This pipeline is illustrated in Fig. 10. The individual steps are numerated from (C1) to (C4). To set up the classification pipeline, we used the python module scikit-learn^51^.

The DRB set and the *validation* set were fed into the classification pipeline. For each classifier in Table 1, the DRB set was used to optimize the classification parameters via a parameter grid search (C1). Here, multiple additional cross-validations were performed, which are not displayed in Fig. 10.

We performed a 144-fold 80%-20% cross-validation split on the DRB set to generate the *training* and *testing* sets (C2). The classifier was trained on *training* with the optimized parameters (C3) and the performance was evaluated on *training*, *testing* and *validation* (C4).

To analyze the model performance, we calculated the following four classification metrics:

- $$\text {accuracy} = \frac{t_p + t_n}{t_p + t_n + f_p + f_n}$$
- $$\text {precision} = \frac{t_p}{t_p + f_p}$$
- $$\text {recall} = \frac{t_p}{t_p + f_n}$$
- $$f_1 = 2 \cdot \frac{\text {Precision} \cdot \text {Recall}}{\text {Precision} + \text {Recall}}$$

Here $t_p$, $t_n$, $f_p$, $f_n$ are the number of true positives, true negatives, false positives and false negatives in the classification. As precision and recall are binary metrics, they were calculated for each material type individually and then averaged.

The classification metrics and errors were calculated over all $25\times 144$ evaluations. For each classification, we generated a confusion matrix normalized along the rows based on the predictions of the classifier.

### Detecting identifying characteristics in PL spectra

We are interested in finding spectral fingerprints of certain sample properties in the data. The properties are supplied as labels for each measurement in the metadata.

We attempt to link individual SDCM signatures or PCs to specific sample properties. As both DR methods are unsupervised, no *validation* set is necessary, nor do we need to re-project the data onto the discovered cluster axes. The data preparation workflow hence only consists of the steps (P1), (P2), (P3a) and (P4) in Fig. 8. PCA and SDCM are applied only once to the data. In contrast to the previous section, we do not aggregate all non-plastic material types into a single label.

In the following, we refer to both SDCM signatures and PCA PCs as *clusters*. To be able to interpret the discovered clusters, we imposed the restriction that for each measurement all data labels must be provided. The dimensions of the data considered are $N_f \times N_m = 1394 \times 1243$.

#### Cluster weights

To match clusters to properties, we needed to determine if a measurement is part of cluster. For each cluster *k* and measurement $m^j$, SDCM provides weights $w^{k,j} \in \big [-1,1\big ]$, which can be used to quantify how strongly a measurement is associated with a cluster *k*. We consider a measurement to be part of a cluster if $|w^{k,j}|\approx 1$, whereas if $|w^{k,j}|\approx 0$ there is no link present. Each cluster has an axis, along which the measurements are clustered, whose median (after median subtraction) is located at 0. This separates the axis into one part with negative weights and one with positive weights.

Since the implementation of PCA does not provide a comparable metric, we needed to define such a quantity for PCs. Let $C^{k, j}_{\text {PCA}}$ be the coefficient of the *j*-th measurement in the *k*-th PC. We defined the PC weight as:

$$\begin{aligned} w^{k,j}_{\text {PCA}} = \frac{C^{k, j}_{\text {PCA}}}{\max _{j\in \mathcal {M}} |C^{k, j}_{\text {PCA}}|} \end{aligned}$$

where $\mathcal {M}$ is the set of all measurements. The interpretations of positive and negative weights are identical to the one previously described for SDCM.

We increased the number of clusters to test for associations to sample properties by dividing each cluster *k* into three subclusters: one which includes all measurements with $w>0$, one with measurements that fulfill $w<0$ and one that is identical to *k*. For SDCM, the weights are closely distributed around either $\pm 1$ or 0. This motivates determining cluster membership of a sample by a threshold $\tau \in \big [0,1\big ]$. We say

$$\begin{aligned} k^- \,\text {contains}\, m^j&\iff w^{k,j} < 0 \quad \text {and} \quad |w^{k,j}| \ge \tau \\ k^+\, \text {contains}\, m^j&\iff w^{k,j} > 0 \quad \text {and} \quad |w^{k,j}| \ge \tau \\ k\, \text {contains}\, m^j&\iff |w^{k,j}| \ge \tau \end{aligned}$$

For the remaining part of this chapter, we refer to all subclusters as $k^*$.

The optimal $\tau$ for both PCA and SDCM can be found empirically by testing the association between subclusters and labels for multiple values. SDCM is fairly robust under different choices of the threshold. Here, $\tau$ can be reliably set to 1. PCA, in contrast, is more sensitive to this choice and performs best when $\tau$ is between 0.05 and 0.4 (see SI for a discussion). The best value for $\tau$ can be determined by optimizing the number of matches at $f_1 \ge 90$ (see below for an explanation for the calculation of $f_1$).

#### Quantifying the association between a subcluster and a set of labels

Let *l* be a label and $k^*$ a subcluster. *l* is associated to $k^*$ if it is likely for a measurement belonging to $k^*$ to have carry the label *l* and vice versa. We can describe this relationship in a contingency table *T* (see Table 5). A strong association should lead to a large number of true positives/negatives relative to the false positives/negatives. Mathematically, we are interested in the *recall* (fraction of measurements carrying *l* that belong to $k^*$) and *precision* (fraction of measurements belonging to $k^*$ that carry *l*) of the contingency table. The $f_1$ score is the harmonic mean of recall and precision and therefore a suitable score to summarize both values. We always have $0 \le f_1 \le 100$ and $f_1 = 100$ for a perfect association with no false positives/negatives. We interpret $f_1$ as a measure of how well *L* matches to $k^*$.

**Table 5:** Contingency table for subcluster–label association.

	$m^j$ carries *l*	$m^j$ does not carry *l*
$m^j\in k^*$	True positives	False positives
$m^j\not \in k^*$	False negatives	True negatives

$m^j \in k^*$ is to be read as *the measurement* $m^j$ *is belongs to* $k^*$.

#### Associating one cluster with one label

We searched for subcluster–label associations by exhaustively calculating $f_1$ for every subcluster and every available label *l*. To do so, we first constructed the set of all theoretically possible labels ${\mathcal {L}}$, which is given by all Cartesian products of all category sets. As the number of all labels generated in this way is much larger than the number of labels that can be realistically recorded experimentally, real labels are often related. For example, if all samples of type “PVC” have the color “red” and all “red” samples are of type “PVC”, then the labels “PVC”, “red” and “PVC, red” are equivalent descriptions of the underlying set of measurements.

We say that two labels, $l_1, l_2 \in {\mathcal {L}}$, are *equivalent*, $l_1 \sim l_2$, if they describe the same set of measurements. We define ${\mathcal {L}}/\sim$ as the set of equivalence classes (ECs) induced by this equivalence relation. As every label of an EC belongs to the same set of measurements, it is sufficient to calculate the $f_1$ score for just one representative of each class.

For every subcluster and every $\text {EC} \in {\mathcal {L}}/\sim$, the $f_1$ score was calculated via the contingency table presented in Table 5. When the number of measurements associated to a label is large relative to total number of measurements, the $f_1$ score may grow large by random association. Hence a *p*-value was calculated with a hypergeometric test. Only matches with $p<0.005$ were kept for further analysis. For every EC, the highest scoring subcluster was chosen.

If an EC contain more than one label, the interpretation of the subcluster–label match is ambiguous. To recover interpretable subcluster–label matches, we chose label representatives from the ECs via the selection rules (A) and (B) as defined in the results. If the selection is not unique, the EC was dismissed.

## Supplementary Information

Supplementary Information.

## Data Availability

The datasets used and analysed during the current study are available from the corresponding author, Prof. Peter Lenz, on reasonable request.
