# Supplementary material for: Identifying plastics with photoluminescence spectroscopy and machine learning
Source: Sci Rep. 2022 Nov 6;12:18840. doi: 10.1038/s41598-022-23414-3 (PMC9637756; doi:10.1038/s41598-022-23414-3)
Supplement: Supplementary file 1 — Supplementary Information. [file 41598_2022_23414_MOESM1_ESM.pdf]

Supplementary Information

Prediction performance for all ML stages

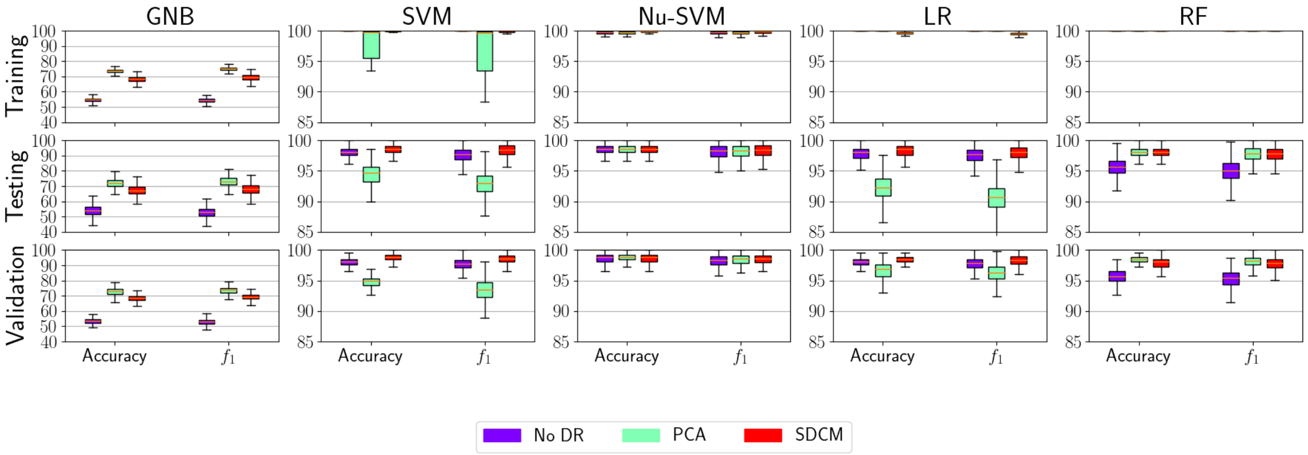

Figure S1. Summary of the prediction performance of ML model for PL-based sample identification during the *Training*, *Testing* and *Validation* stage. no DR, SDCM and PCA refer to the preprocessing of the input data. The accuracy and the f_1 score are presented as box plots.

Associating one cluster with multiple labels

In the main text we described a straightforward approach to test associations between clusters and sample properties, by exhaustively comparing the f_1 score among all available clusters and labels.

However, there are factors limiting this method's applicability:

- In principle, all ECs have to be tested. The number of ECs may grow exponentially depending on the variability of data, and number of label categories.
- A cluster might correspond to a combination of different labels. For example, if several different colors originate from a chemical composition which expresses itself in the measured spectrum, a cluster may match well with *all* measurements carrying any one of those colors, but it may match badly when testing each color individually.

Therefore, we are interested in a procedure to test the association of a cluster with a *combination* of labels. As the number of all label combinations is much larger than the number of labels, this procedure needs to be efficient and must not require an exhaustive testing of all label combinations.

Our proposed procedure works as follows:

1. Split the data into minimal label "building blocks".
2. Iteratively combine the building blocks until the f_1 score of the combination with respect to a cluster is maximized

The label building blocks are generated by drawing each label from all available categories simultaneously (e.g. the label "plastic, PVC, red, supermarket, sample #5" drawn from the categories «is plastic?, origin, color, type, sample ID»). By construction, each measurement carries only one such label. Thus, any general property can be expressed as a combination of any of the available building blocks. For each cluster, the f_1 score is maximized by a greedy optimization process.

Results Fig. S2 shows the distribution of cluster-to-combination matches over f_1 for PCA and SDCM. The weight threshold values τ which determine the cluster membership were chosen to optimize the number of combinations that score with $f_1 \geq 90$. We find that both DR methods achieve several matches at high f_1 scores, with only SDCM achieving 10 *perfect* matches at $f_1 = 100$. Fig. S3 shows scatter plots where each cluster f_1 score is plotted against its combination size for PCA and SDCM and their marginal distributions. As PCA produces far more subclusters than SDCM, more unique combinations can be detected. However, the bulk of these are concentrated at low f_1 scores, and not suitable for interpretation. The combinations with $f_1 \geq 80$ are distributed in a broad range across the size of label combinations N.

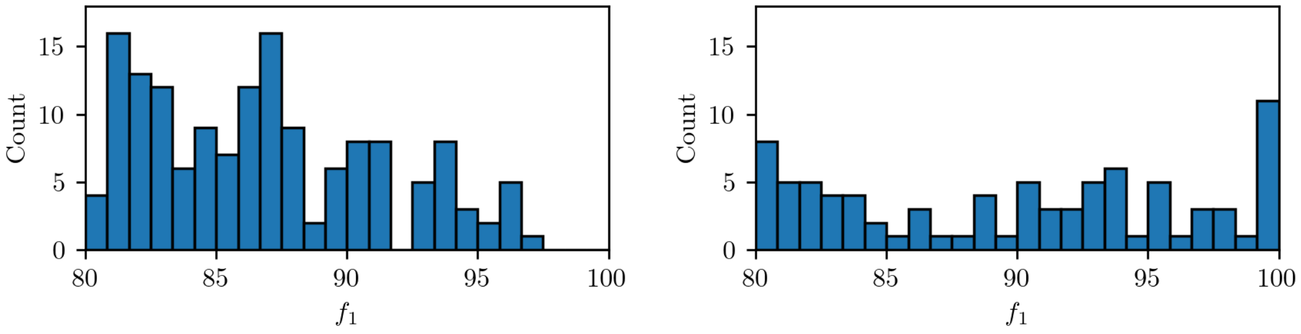

(a) PCA

(b) SDCM

Figure S2. Distribution of f_1 scores for PCA and SDCM in the range $80 \leq f_1 \leq 1$.

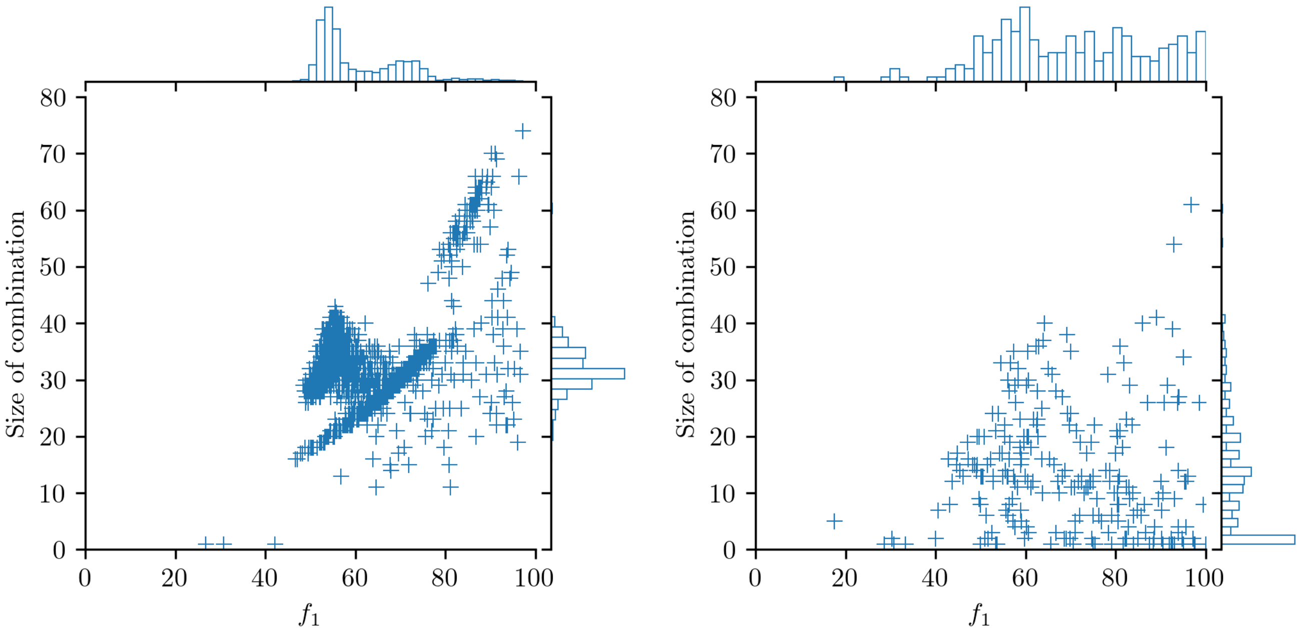

(a) PCA

(b) SDCM

Figure S3. Scatter plot of f_1 score against combination size for PCA and SDCM with their marginal distributions.

While the smaller number of subclusters in SDCM lead to an overall smaller number of matches, the marginal f_1 distribution is shifted towards higher f_1 values compared to PCA. The high scoring matches at small N correspond to our previous observation that SDCM subcluster can be identified with the spectral features of individual samples. However, we now also detect several larger combinations at sizes $10 \leq N \leq 60$, which may be attributed to a more general physical interpretation. This is in contrast to the previous method, where such general interpretations were rare.

We illustrate the dependence of our results on the weight threshold parameter τ by displaying scatter plots for different choices of τ in Fig. S4. We see that the results for SDCM are far more robust against variations of τ, maintaining its ability of finding small and medium-sized high scoring combinations for a large range of values.. The results for PCA vary strongly not only in number of detected combinations, but also in their distribution along the size and f_1 axes.

Methods Every label l is associated with a set of measurements and every measurement m_j can be given an unambiguous maximum label l_j by drawing l_j from all available categories. As a result, l can be constructed as the set union of all l_j "building blocks". The same is true for any *combination* of labels.

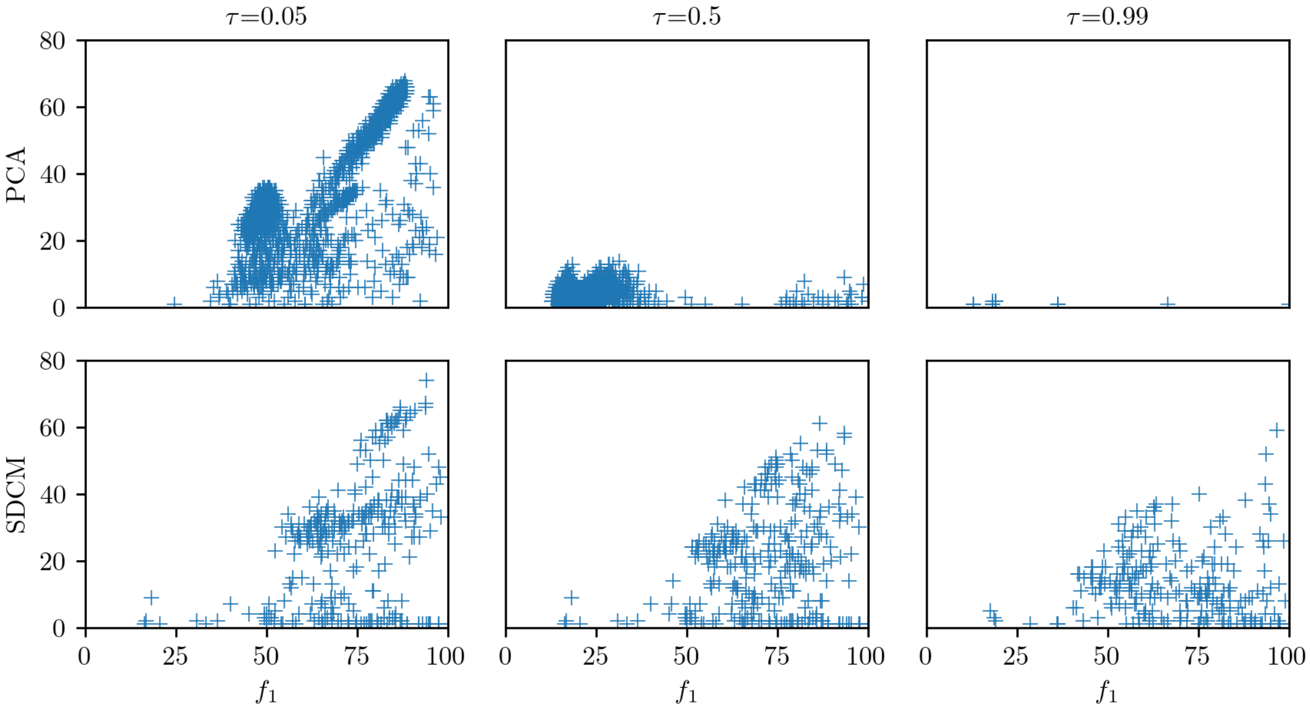

Figure S4. Qualitative overview of robustness of results under different choices of weight threshold parameter τ for PCA and SDCM.

We produced the set of building blocks $\mathscr{B}$ by drawing them from the largest set of categories, i.e. «is plastic, type, origin, color, sample ID». Every measurement belongs to only a single element in $\mathscr{B}$. Any label (or label combination) can be described as a binary vector $\vec{l}$, $l_i \in \{0,1\}$, where $i \in 1,\ldots,|\mathscr{B}|$ and $l_i = 1$ denotes membership of the i-th building block in the combination.

As the number of possible building block combinations is $2^{|\mathscr{B}|}$, calculating f_1 for every k^* and combination is computationally infeasible. Instead, we calculated the optimal combination with a greedy optimization process:

We defined the cost function as

$$C(p,r,r_1,\ldots,r_n) = \min(p,r) + \min_i(r_i)$$

where p and r are the precision and recall of $\vec{l}$ with respect to the subcluster and $r_1,\ldots,r_n$ are the recalls of each individual l_i taking part in the combination. For a given k^*, we initialized $\vec{l}$ as the zero vector. In an iterative process, we successively flipped every element in l_i and calculated p,r,r_i and $C(p,r,r_1,\ldots,r_n)$. To avoid optimization into the trivial case where all $l_i = 1$, we calculated C' from $\vec{l}'$, where $\vec{l}'$ is the binary inverse of $\vec{l}$. For a perfect match, we have $C = C'$. If $\min(C,C')$ was larger than in the previous iteration, the new state was adopted. If $\vec{l}$ did not change for $2|\mathscr{B}|$ iterations, it was accepted as the final building block membership array. Finally, a p-value was calculated from a hypergeometric test. Only subcluster matches with $p \leq 0.005$ were kept for further analysis. If the same $\vec{l}$ was derived for multiple subclusters, the highest scoring subcluster was chosen and all others discarded. We additionally checked whether $\vec{l}$ is the binary inverse of another optimal $\vec{l}'$, to remove redundancies. In such a case, the higher scoring combination would be preferred. This, however, never occurred in our analysis.
